# Mycovorax composti gen. nov., sp. nov., a member of the family Chitinophagaceae isolated from button mushroom compost

**DOI:** 10.1099/ijsem.0.006496

**Published:** 2024-08-27

**Authors:** Meghann Thai, Tina L. Bell, Michael A. Kertesz

**Affiliations:** 1 School of Life and Environmental Sciences, The University of Sydney, Sydney, NSW, 2006, Australia

**Keywords:** *Agaricus bisporus*, Chitin, compost, Phase II, thermophiles

## Abstract

Two Gram-stain-negative, aerobic, rod-shaped, orange-coloured bacterial strains, designated strain C216^T^ and strain M2295, were isolated from mature mushroom compost from composting facilities in Victoria and South Australia, Australia, respectively. External structures such as flagella or pili were not observed on the cells under scanning electron microscopy. Optimal growth was found to occur at 45 °C, at pH 7.25 and in the absence of NaCl on Emerson’s 350 YpSs medium. The genome sequence of strain C216^T^ was 3 342 126 bp long with a G+C content of 40.5 mol%. Functional analysis of the genome of strain C216^T^ revealed genes encoding chitinolytic and hemi-cellulolytic functions, with 166 predicted genes associated with carbohydrate metabolism (8.9% of the predicted genes). These functions are important for survival in the mushroom compost environment, which is rich in hemicelluloses. No antibiotic resistance genes were found in the genome sequence. The major fatty acids of strain C216^T^ were iso-C_15 : 0_ (56.7%), iso-C_17 : 0_ 3-OH (15.6%), C_16 : 1_* ω*7*c*/iso-C_15 : 0_ 2-OH (7.3%) and iso-C_15 : 1_ G (6.1%). The only respiratory quinone was MK-7. The major polar lipid of strain C216^T^ was phosphatidylethanolamine, but three unidentified phospholipids, four unidentified aminophospholipids/aminolipids and one unidentified glycolipid were also detected. Phylogenetic analysis based on proteins encoded by the core genome (bac120, 120 conserved bacterial genes) showed that strain C216^T^ forms a distinct lineage in the family *Chitinophagaceae* and that the closest identified relative is *Niabella soli* (69.69% ANI). These data demonstrate that strain C216^T^ represents a novel genus and novel species within the family *Chitinophagaceae*, for which we propose the name *Mycovorax composti*. The type strain is C216^T^ (=DSM 114558^T^=LMG 32998^T^).

## Introduction

Mushroom compost is a highly selective substrate that is used to support the growth of button mushrooms (*Agaricus bisporus*). It is produced from wheat straw, poultry manure and gypsum, which are transformed into compost by the bacteria and fungi that colonize these feedstocks. Composting starts with wetting the wheat straw for a period of 3–10 days, followed by addition of poultry manure and gypsum [1]. The mixture is then turned periodically to homogenize and aerate the compost over 6–14 days (phase I) [1,2]. During phase I, heat is rapidly generated by the microbial activity, and peak compost temperatures are as high as 80 °C [3]. This is followed by phase II, in which the partially fermented compost is pasteurized in enclosed tunnels at 58–60 °C for 8 h [1] and then conditioned by decreasing compost temperatures slowly from 58 to 45 °C over 4 days [4,5]. The microbial dynamics during conditioning are of particular interest, as 50–60% of the compost carbohydrates are degraded during this period [6] and incorporated into microbial biomass or respired. When *A. bisporus* is added to the compost it exploits the bacterial and fungal biomass of this community as a key nutrition source during mycelial proliferation [7]. *Chitinophagaceae* have been found in various compost systems [5,8,10], and this family is one of the more abundant taxa found during the conditioning stage in phase II compost [5].

The family *Chitinophagaceae* is part of the phylum *Bacteroidota* and was first described by Kämpfer *et al.* [11]. The type genus is *Chitinophaga*, which was first described by Sangkhobol and Skerman [12] as a chitinolytic myxobacterium, but was later transferred to the family *Chitinophagaceae* [11]. More than 40 other genera belong to the family *Chitinophagaceae*, including *Compostibacter* [13], *Filimonas* [14], and *Pseudocnuella* [15]. Cells from genera in the family *Chitinophagaceae* contain menaquinone MK-7 as the major quinone and the major fatty acids are iso-C_15 : 0_, iso-C_17 : 0_ 3-OH, and iso-C_15 : 1_G [11]. Cells are usually thin, rod-shaped, and non-motile; however, swarming motility may be observed [11]. They are usually mesophilic and aerobic or facultatively anaerobic, and some members of this family have limited fermentative capabilities [11].

Two bacterial strains, designated C216^T^ (=DSM 114558^T^=LMG 32998^T^) and M2295 (=DSM 114559=LMG 32997), were isolated from button mushroom compost at the end of phase II. These strains demonstrated chitinolytic activity *in vitro*, and during growth in laboratory media they were antagonistic to *Mycothermus thermophilus*, a thermophilic cellulolytic ascomycete fungus that is dominant in mushroom compost.

## Isolation and ecology

End-phase II compost was collected from commercial mushroom compost yards in Victoria and South Australia, Australia. Compost samples (approximately 1 g) were suspended in 9 ml of Reasoner's 2A (R2A) broth (HiMedia Laboratories) and incubated at 40 °C for 48 h with shaking (200 r.p.m.). Samples were then plated on R2A agar plates (Oxoid), and round orange colonies with entire margins were purified by subculturing on R2A agar (45* °*C, 48 h). The bacterial strain isolated from mushroom compost from the compost yard in Victoria was designated C216^T^, and the bacterial strain isolated from the compost yard in South Australia was designated M2295. Both strains were routinely cultured on Emerson’s YpSs agar [16] at 45 °C and preserved at −80 °C in glycerol suspension (25% v/v).

Prior to isolation, this bacterial taxon had been detected in a comprehensive study of microbial succession during mushroom composting at a commercial compost yard in New South Wales, Australia [5], using 16S diversity profiling with Illumina sequencing. The organism was part of a stable bacterial community that developed in phase II mushroom compost, dominated by *Pseudoxanthomonas*, *Steroidobacter*, and the novel genus from the family *Chitinophagaceae* reported here. The main fungal taxon associated with these bacteria was *M. thermophilus*, which is dominant in Phase II mushroom compost [5,17] and has been extensively studied for its role in promoting the growth of *A. bisporus* [18]. When strain C216^T^ was grown in co-culture with *M. thermophilus*, the bacterium exhibited chitinolytic activity on the hyphal tips of the fungus (see below), suggesting that it plays an important ecological role in mushroom compost.

## 16S rRNA phylogeny

Genomic DNA was extracted using the CTAB method, according to the method of Wilson [19]. Briefly, late exponential phase bacterial cells were harvested and resuspended in Tris-ethylenediaminetetraacetic acid (TE) buffer (10 mM Tris-HCl, 1 mM EDTA, pH 8.0; 100 µl) with 1 mg ml^−1^ lysozyme (Sigma-Aldrich) and incubated at 37 °C for 30 min without agitation. Hexadecyltrimethylammonium bromide (CTAB) extraction buffer (2% CTAB (w/v), 100 mM Tris-HCl, 20 mM EDTA, 1.4 M NaCl; 500 µl) was added and the mixture was incubated at 50 °C for 60 min without agitation. Chloroform:isoamyl alcohol (24 : 1) was added in equal volume and the solution was emulsified before centrifuging at 14 000 *g* for 10 min to separate the two phases. The top aqueous phase (400 µl) was transferred to a fresh tube and cold isopropanol (320 µl) was added. The precipitated DNA was collected by centrifugation (20 000 *g* for 20 min) and washed twice with 80% (v/v) ethanol. The DNA obtained was resuspended in TE buffer (pH 8.0) and stored at −20 °C.

**Fig. 1.**
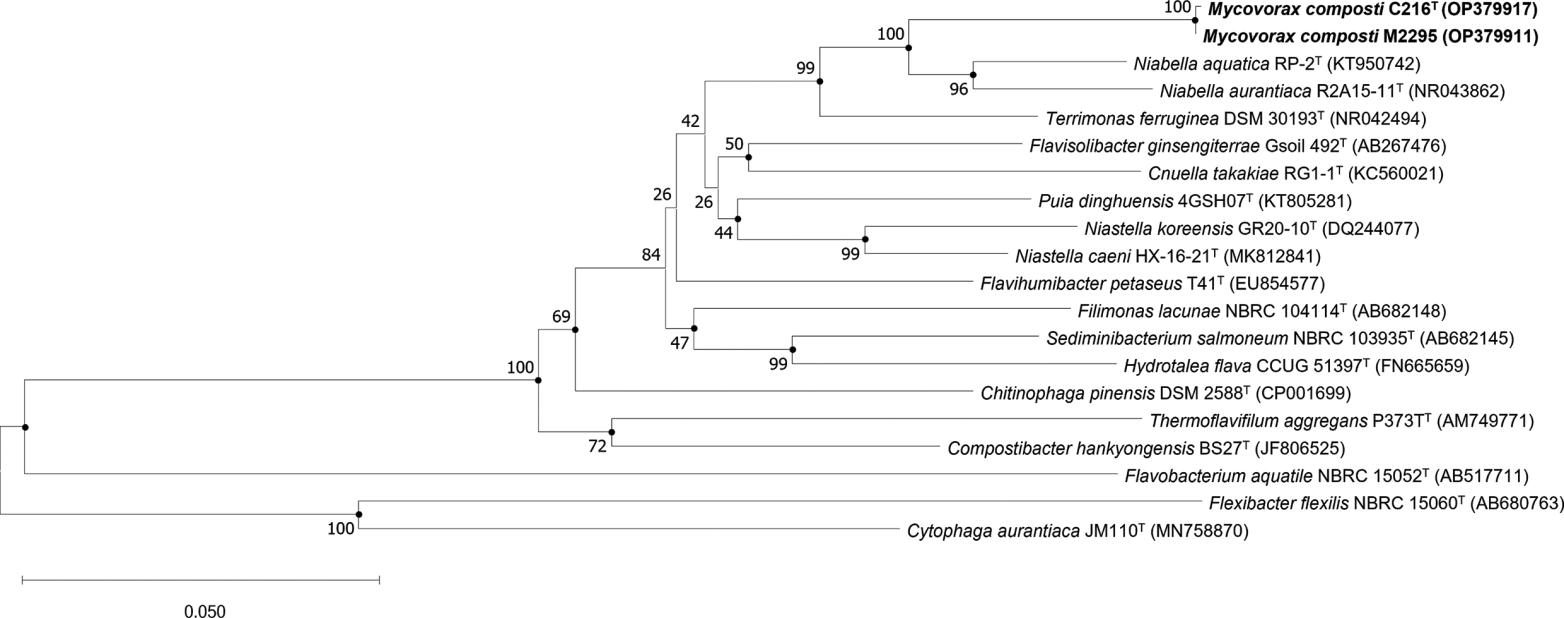
Neighbour-joining phylogenetic tree for *Mycovorax composti* strains C216^T^ and M2295, and related strains based on partial 16S rRNA gene sequences. Filled circles (●) indicate nodes that were the same in the maximum-likelihood tree. Numbers at nodes indicates the percentages of bootstrap support based on 1000 replications. Bar, 0.05 substitutions per nucleotide position. *Flavobacterium aquatile* NBRC 15052^T^, *Flexibacter flexilis* NBRC 15060^T^ and *Cytophaga aurantiaca* JM110^T^ were used as outgroups.

The full 16S rRNA gene sequence of strain C216^T^ was obtained from the genome sequence (see below). The partial 16S rRNA gene sequence of strain M2295 was determined by Macrogen Inc. (Seoul, Republic of Korea) following their standard protocol. Pairwise alignment of the 16S rRNA gene sequences of strains C216^T^ and M2295 showed that they were 99.93% identical. 16S rRNA gene sequences of closely related genera were obtained from the National Center for Biotechnology Information (NCBI) [20]. The 16S rRNA gene sequences were aligned using Clustal W [21], and a phylogenetic tree was reconstructed using the software package mega-X (version 10.2.2) [22]. Distances were determined using the Kimura two-parameter model and clustering was done with the neighbour-joining and maximum-likelihood methods. The reliability of the trees obtained was confirmed using bootstrap values based on 1000 replicates. The neighbour-joining tree (Fig. 1) revealed that strain C216^T^ was closely related to members of the family *Chitinophagaceae* within the phylum *Bacteroidota*, with strains C216^T^ and M2295 forming a separate branch next to the genera *Niabella* and *Terrimonas.* The maximum-likelihood tree showed similar topology. Pairwise comparison of the full 16S rRNA gene sequence of strain C216^T^ (1 526 bp) with those of *Niabella aquatica* and *Terrimonas ferruginea* revealed 92.0–93.5 % 16S rRNA gene sequence similarity to the type strains of these species.

## Genome features and phylogeny

Whole genome sequencing of the C216^T^ genomic DNA was done by the Microbial Genome Sequencing Center (Pittsburgh, USA), using their standard Illumina protocols (NextSeq 2000; 2×150 bp). Long-read sequencing was done using an R10.4.1 flow cell on minION apparatus (Oxford Nanopore Technology). The resulting FastQ files were analysed using the Galaxy software package [23]. Long-read sequence data were filtered and trimmed using Porechop (version 0.2.4) [24] and filtlong (version 0.2.1) [25], with 90% adapter sequence similarity and 500 bp minimum sequence length, respectively. Trimmed reads were assembled using Flye (version 2.9.1) [26]. Genome closure was achieved by aligning the Illumina sequencing reads to the draft genome using BWA-MEM (version 0.7.17.2) [27] and the genome was polished using pilon (version 1.20.1) [28]. Genome annotation was done in Galaxy using Barrnap and Prokka [29,30]. Functional analysis of the genome of strain C216^T^ was done using eggNOG (version 5.0.2) [31] available in the Galaxy software package, and rast with the RASTtk annotation pipeline [32,34]. DNA G+C content of strain C216^T^ was determined from the genome sequence.

The complete genome size of strain C216^T^ was 3 342 126 bp. DNA G+C content of strain C216^T^ was 40.5 mol%. The genome was assembled into one contig which contained 2886 predicted genes, including 2839 coding DNA sequences, six rRNA regions, 40 tRNA regions and one tmRNA region. Barrnap [30] annotation revealed that the genome of strain C216^T^ contained two identical copies of the 16S rRNA gene, 23S rRNA gene and 5S rRNA genes. There were no antibiotic resistance genes identified in the genome. A relatively high proportion (54.1%) of the protein-coding genes could be assigned putative functions by Prokka [29], while the remaining genes were annotated as hypothetical proteins. The genome sequence is available with accession number CP144143. Pairwise analysis of the whole genome of strain C216^T^ was done using the Type Strain Genome Server [35], and revealed that *Myroides aquimaris* CGMCC 1.10825^T^ was the most closely related strain, with 34.6% similarity in terms of digital DNA–DNA hybridization (dDDH; formula d_4_). *Niabella aurantiaca* DSM 17617^T^ had only 19.4% pairwise similarity with strain C216^T^ when comparing dDDH (d_4_).

Both strains were isolated from wheat straw compost, and therefore genes that are responsible for cellulose and hemi-cellulose degradation were of particular interest. Xylan is a type of hemicellulose commonly found in wheat straw, and is constructed of a *β*-1,4-xylose backbone with *α*-l-arabinofuranosyl, acetyl and 4-O-methylglucuronyl branched units [6,36]. Analysis of the C216^T^ genome revealed putative genes encoding endo-1,4-*β*-xylanase (EC 3.2.1.8), *β*-d-xylosidase (EC 3.2.1.37), *α*-l-arabinofuranosidase (EC 3.2.1.55) and acetylxylan esterase (EC 3.1.1.72), suggesting that strain C216^T^ degrades xylan found in wheat straw. No genes for cellulose degradation were found. The functional genes found in strain C216^T^ therefore suggest that the strain is highly specialized for the breakdown of hemi-celluloses in the thermophilic, wheat straw compost environment in which it is found. Further genome features for strain C216^T^ and its six closest phylogenetic neighbours are provided in Tables S1 and S2.

Another feature of strain C216^T^ was its interaction with *Mycothermus thermophilus*, which it antagonized *in vitro* by breaking down the fungal hyphae (Fig. S1, available in the online version of this article). This is likely due to the presence of chitin degrading enzymes since chitin is the main structural polymer in fungal cell walls. No genes encoding endo-chitinases (EC 3.2.1.14) were found in the genome of strain C216^T^, but predicted genes for exo-*N*-acetylglucosaminidases (EC 3.2.1.52) were present. Exo-*N*-acetylglucosaminidases have been shown to cleave chitin oligosaccharides such as chitobiose and chitotriose [37].

rast annotation software assigned 239 groups of functional genes, known as subsystems, from the 2922 protein coding sequences within the genome of C216^T^. The highest number of assigned genes was associated with biosynthesis and metabolism of amino acids (178), followed by carbohydrate subsystems (166) and protein metabolism (124), respectively (Fig. S2). Within the carbohydrate subsystem, several genes involved with chitin and xylose utilization were annotated. COG analysis of strain C216^T^ proteins showed similar annotations to the rast annotation. The majority of the functional genes were assigned to unknown functions (22%), while 8.9% of the functional genes were associated with carbohydrate metabolism. Several proteins associated with chaperones and heat tolerance were also found in the C216^T^ genome, allowing it to survive the high temperatures in phase II mushroom compost.

**Fig. 2.**
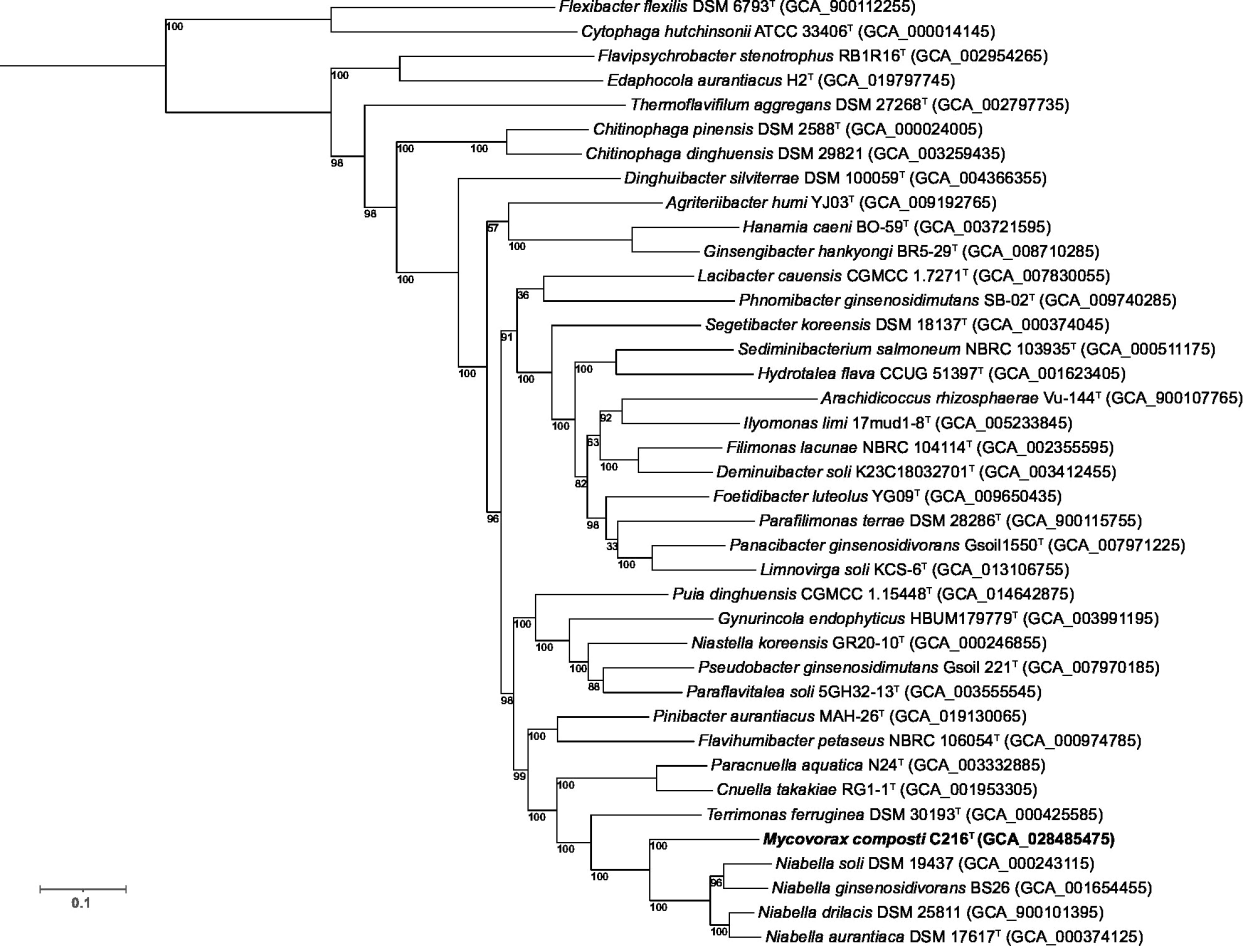
Maximum-likelihood phylogenetic tree of the famiy *Chitinophagaceae* based on concatenated amino acid sequences of 120 conserved bacterial proteins. *Cytophaga hutchinsonii* ATCC 33406^T^ and *Flexibacter flexilis* DSM 6793^T^ were used as outgroups. Numbers at nodes are percentages of bootstrap support based on 1000 replications. Scale bar, 0.1 substitutions per nucleotide position.

CAZy analysis from the eggNOG [31] package in Galaxy [23], showed that 127 proteins were associated with glycoside hydrolases (GHs), glucosyltransferases (GTs), carbohydrate binding modules (CBMs), carbohydrate esterases (CE) and polysaccharide lyases (PLs) families in strain C216^T^. Most of these proteins were associated with GH families (57%), followed by GT families (28%) and CE, CBM and PL families (6, 4, 4%, respectively). No proteins were assigned to the auxiliary activities (AA) family. Most of the enzymes in the GH family were associated with cleavage of *β*-1,4-linked glycosidic bonds in a broad range of carbohydrates. The genome of strain C216^T^ was compared with the genome of 36 type species within the family *Chitinophagaceae*, using genome sequences sourced from NCBI [20]. A phylogenetic tree based on 120 concatenated conserved bacterial protein amino acid sequences [38] was reconstructed using the dataset provided by GTDB-tk (version 2.2.2, release 207.2) [39] on Galaxy Australia [23]. A maximum-likelihood tree using these core sequences was reconstructed in Galaxy using FASTTree (version 2.1.10) using model LG+CAT [40]. The phylogenetic tree shows that strain C216^T^ formed a separate branch between the genera *Niabella* and *Terrimonas*, clearly indicating that strain C216^T^ represents a novel genus within the family *Chitinophagaceae* (Fig. 2).

Average nucleotide identity (ANI) values were calculated using OrthoANI, the online tool from EzBioCloud [41]. Average amino acid identity (AAI) values were calculated with the online tool provided by the Environmental Microbial Genomics Laboratory [42]. The ANI and AAI values between strain C216^T^ and its most closely related species were 69.5 and 64.7%, respectively (Table 1). These values are well below the cutoff value for ANI that has been proposed to delineate species (95–96%) and the AAI value (74–76%) used to distinguish genera [43,44].

**Table 1.** AAI and ANI values between strain C216^T^ and other related type species in the family *Chitinophagaceae*

Strain	GenBank accession no.	AAI (%)	ANI (%)
*Niabella ginsenosidivorans* BS26^T^	GCA_001654455.1	64.78	69.52
*Niabella aurantiaca* DSM 17617^T^	GCA_000374125.1	64.76	69.42
*Niabella soli* DSM 19437^T^	GCA_000243115.3	64.72	69.69
*Niabella drilacis* DSM 25811^T^	GCA_900101395.1	64.15	69.46
*Terrimonas ferruginea* DSM 30193^T^	GCA_000425585.1	59.85	68.15
*Lacibacter cauensis* CGMCC 1.7271^T^	GCA_007830055.1	56.26	67.89
*Pinibacter aurantiacus* MAH-26^T^	GCA_019130065.1	56.06	68.09
*Paracnuella aquatica* N24^T^	GCA_003332885.2	55.94	67.66
*Paraflavitalea soli* 5GH32-13^T^	GCA_003555545.1	55.82	67.84
*Pseudobacter ginsenosidimutans* Gsoil 221^T^	GCA_007970185.1	55.76	67.21
*Agriteriibacter humi* YJ03^T^	GCA_009192765.1	55.65	67.66
*Parafilimonas terrae* DSM 28286^T^	GCA_900115755.1	55.41	67.78
*Hydrotalea flava* CCUG 51397^T^	GCA_001623405.1	55.31	68.04
*Panacibacter ginsenosidivorans* Gsoil1550^T^	GCA_007971225.1	55.23	67.9
*Cnuella takakiae* RG1-1^T^	GCA_001953305.1	55.22	67.54
*Niastella koreensis* GR20-10^T^	GCA_000246855.1	55.21	67.64
*Foetidibacter luteolus* YG09^T^	GCA_009650435.1	55.00	67.89
*Sediminibacterium salmoneum* NBRC 103935^T^	GCA_000511175.1	54.93	68.13
*Ilymonas limi* 17mud1-8^T^	GCA_005233845.1	54.92	67.92
*Segetibacter koreensis* DSM 18137^T^	GCA_000374045.1	54.8	67.47
*Limnovirga soli* KCS-6^T^	GCA_013106755.1	54.7	67.97
*Flavihumibacter petaseus* NBRC 106054^T^	GCA_000974785.1	54.69	67.2
*Phnomibacter ginsenosidimutans* SB-02^T^	GCA_009740285.1	54.59	67.58
*Deminuibacter soli* K23C18032701^T^	GCA_003412455.1	54.53	67.8
*Gynurincola endophyticus* HBUM179779^T^	GCA_003991195.1	54.49	67.63
*Hanamia caeni* BO-59^T^	GCA_003721595.1	54.45	67.1
*Filimonas lacunae* NBRC 104114^T^	GCA_002355595.1	54.39	67.71
*Ginsengibacter hankyongi* BR5-29^T^	GCA_008710285.1	54.34	67.12
*Puia dinguensis* CGMCC 1.15448^T^	GCA_014642875.1	54.07	67.07
*Arachidicoccus rhizosphaerae* Vu-144^T^	GCA_900107765.1	53.05	66.85
*Dinghuibacter silviterrae* DSM 100059^T^	GCA_004366355.1	52.95	66.44
*Chitinophaga caeni* 13^T^	GCA_002557795.1	52.82	67.01
*Chitinophaga pinensis* DSM 2588^T^	GCA_000024005.1	52.59	66.46
*Chitinophaga dinghuensis* DSM 29821^T^	GCA_003259435.1	52.02	66.55
*Thermoflavifilum aggregans* DSM 27268^T^	GCA_002797735.1	51.08	66.37
*Edaphocola aurantiacus* H2^T^	GCA_019797745.1	49.41	66.62
*Flavipsychrobacter stenotrophus* RB1R16^T^	GCA_002954265.1	48.71	66.81

## Physiology and chemotaxonomy

Cell morphology was determined using cells grown in Emerson’s YpSs broth for 24 h at 45 °C and viewed using a scanning electron microscope (Sigma VP HD, Zeiss). Growth was tested at different temperatures (30, 37, 40, 45, 50, 55 and 60 °C) and at pH values between pH 4.0 and 9.0 (in increments of 0.25 pH units) for 72 h. Salt tolerance was tested in R2A liquid medium supplemented with 0, 1, 2, 3, 4, 5, 6 and 9% (w/v) NaCl. Motility was assessed using the hanging drop method using a light microscope at ×400 magnification. Gram staining was done according to Smibert [45]. Catalase activity was determined by observing bubble production in 3% (v/v) H_2_O_2_. Oxidase activity was determined by swatching a single bacterial colony (grown on R2A agar for 48 h at 45 °C) onto paper saturated with 1% (w/v) tetramethyl-para-phenylenediamine HCl. The presence of flexirubin-type pigments was tested by flooding the plate with 20% KOH (w/v) and observing colour change [46]. Biochemical characterization was done using the API 20NE (bioMérieux) kit, following the manufacturer’s instructions.

The cellular fatty acid profile was determined using cells grown in Emerson’s YpSs broth for 24 h at 45 °C. Analysis of cellular fatty acids was done by the Identification Service, Leibniz-Institut DSMZ – Deutsche Sammlung von Mikroorganismen und Zellkulturen GmbH, Braunschweig, Germany. Cellular fatty acids were analysed after conversion into fatty acid methyl esters by saponification, methylation, and extraction according to the protocol of Sherlock Microbial Identification System (midi, Microbial ID, Delaware USA). Analysis of respiratory quinones were carried out by DSMZ services, Leibniz-Institut DSMZ – Deutsche Sammlung von Mikroorganismen und Zellkulturen GmbH, Braunschweig, Germany.

Polar lipids for strain C216^T^ were determined from fresh cells and visualized using two-dimensional thin layer chromatography on silica gel 60 according to Nguyen and Kim [47]. Total polar lipids were detected using 5% ethanolic phosphomolybdic acid, aminolipids were detected with ninhydrin, phospholipids were detected with bromthymol blue, and glycolipids were detected with diphenylamine [48].

**Fig. 3.**
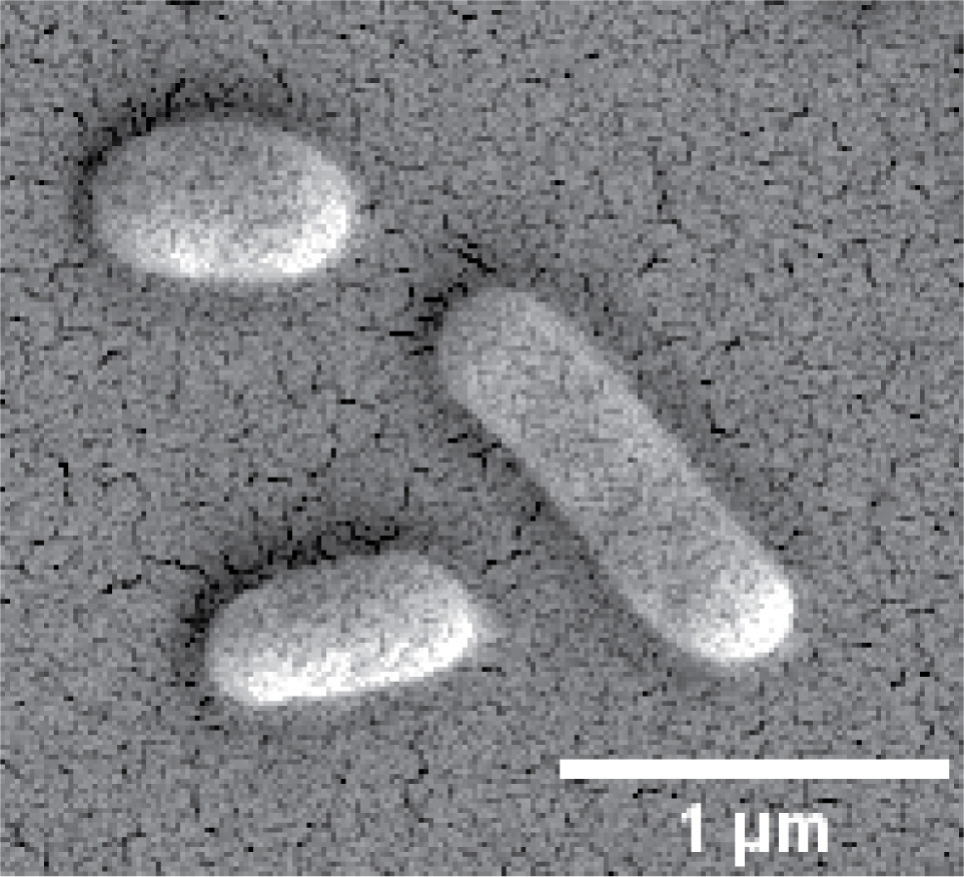
Scanning electron microscope image of C216^T^ cell morphology. The longer cell on the right is an elongated cell that is about to divide. No flagella or other external structures can be seen on the cell.

Cells of strains C216^T^ and M2295 were Gram-stain-negative, short rod shaped, aerobic, non-motile and non-spore forming. Colonies of strains C216^T^ and M2295 grown on R2A, TSA (tryptic soy agar) and Emerson’s YpSs agar at 45 °C for 48 h were circular, convex, opaque, and orange in colour. Growth did not occur on MacConkey agar. Cells were approximately 0.8–1.2 µm long and 0.4–0.5 µm wide (Fig. 3). Growth on R2A medium occurred at 37–55 °C and at pH 6.5–8.0. Tolerance to NaCl was 0–2% (w/v). Dark red to brown flexirubin-type pigments were observed when the plate was flooded with 20% KOH (w/v). Phenotypic and chemotaxonomic characteristics that differentiate this strain from closely related taxa are listed in Table 2.

**Table 2.** Phenotypic and chemotaxonomic characteristics of *Mycovorax composti* strains C216^T^ and M2295, and type strains of closely related genera in the family *Chitinophagaceae* Strains: 1, C216^T^; 2, M2295; 3, *Niabella aurantiaca* R2A15-11^T^; 4, *Hydrotalea flava* CGUG 51397^T^; 5, *Compostibacter hankyongensis* BS27^T^; 6, *Terrimonas ferruginea* ATCC 13524^T^; 7, *Niastella koreensis* GR20-10^T^; 8, *Chitinophaca pinensis* UQM 2034^T^. +, Positive; −, negative; w, weak; nd, no data available.

Characteristic	1	2	3	4	5	6	7	8
Colony colour	Orange	Orange	Orange	Orange-yellow	Milky white	Salmon red	Light yellow	Yellow
Cell morphology	Short rod	Short rod	Rods	Thin rods	Short rod	Rods	Filamentous	Rod
Gram stain	−	−	−	−	−	−	−	−
Relationship to O_2_	Aerobic	Aerobic	Aerobic	Aerobic	Aerobic	Strictly aerobic	Aerobic	Aerobic
Gliding motility	−	−	−	−	−	−	+	+
Growth temperature range (°C)	37–55	37–55	10–30	20–37	15–37	10–37	10–37	12–37
Optimum growth temperature (°C)	40–50	40–50	25–30	nd	30	25–32	nd	24
pH range for growth	6.5–8.0	6.5–8.0	5.0–8.0	nd	6.0–9.0	nd	5.0–8.0	4–10
Salinity range for growth (%)	0–2.0	0–2.0	0–3.0	0–4.0	0–1.5	0–1.0	nd	0–1.5
Flexirubin production	+	+	+	−	−	nd	−	nd
Catalase	w	w	+	+	+	w	−	+
Oxidase	+	+	−	+	−	+	−	−
Indole	−	−	+	nd	−	−	−	−
H_2_S	−	−	nd	−	nd	−	nd	−
Hydrolysis of:								
Starch	+	+	−	nd	−	+	−	−
Chitin	+	+	−	nd	nd	−	+	+
CM-Cellulose	−	−	−	nd	−	nd	+	−
Xylan	−	−	−	nd	−	nd	nd	nd
Casein	−	−	+	nd	−	nd	+	+
Gelatin	−	−	−	nd	−	+	+	+
Urea	−	−	−	nd	−	−	−	+
Voges–Proskauer	−	−	nd	nd	nd	+	nd	nd
Nitrate reduction	−	−	−	−	+	+	−	−
DNA G+C content (mol%)	40.5	nd	45.0	42.0	53.0	48.9	45.8	44.6
Assimilation of sugars:								
Amygdalin	−	−	nd	−	nd	nd	nd	nd
Arabinose	+	+	nd	−	−	nd	nd	nd
Cellobiose	+	+	nd	−	nd	+	nd	nd
Glucose	+	+	−	−	+	+	−	+
Maltose	+	+	nd	−		+	nd	nd
Mannose	−	−	nd	−	+	+	nd	nd
Melibiose	−	−	nd	−	+	+	nd	nd
Mannitol								
Rhamnose	−	−	nd	−	nd	+	nd	nd
Sorbitol	−	−	nd	−	+	−	nd	nd
Sucrose	−	−	nd	−	+	−	nd	+
Xylose	+	+	nd	nd	nd	+	nd	nd
*N*-Acetyl-glucosamine	+	+	nd	nd	nd	nd	nd	nd

Data in columns 3–8 were taken from [11,13,49]((; (; (; (; [] and [].+, Positive; -, negative; w, weak; , no data available

The DNA G+C content of strain C216^T^ was 40.5 mol%, which was less than that of *Niabella* species (45.0 mol%) and *Terrimonas* species (48.9 mol%) (Table 2). Menaquinone-7 (MK-7) was detected as the only respiratory quinone. The major fatty acids (>5 %) identified in strain C216^T^ were iso-C_15 : 0_ (56.7%), iso-C_17 : 0_ 3-OH (15.6%), summed feature 3 (C_16 : 1_* ω*7*c* and/or iso-C_15 : 0_ 2-OH, 7.3%) and iso-C_15 : 1_ G (6.1%). The total fatty acid profile of strain C216^T^ in comparison to closely related genera is summarized in Table 3. Much higher levels of fatty acid iso-C_15 : 0_ (56.7%) clearly distinguished strain C216^T^ from *Niabella* and *Terrimonas* species and contribute to the thermotolerant/thermophilic nature of this strain.

**Table 3.** Fatty acid profile of strain C216^T^ and related type strains of genera in the family *Chitinophagaceae* Strains: 1, C216^T^; 2, *Niabella aurantiaca* R2A15-11^T^; 3, *Hydrotalea flava* CGUG 51397^T^; 4, *Compostibacter hankyongensis* BS27^T^; 5, *Terrimonas ferruginea* ATCC 13524^T^; 6, *Niastella koreensis* GR20-10^T^; 7, *Chitinophaga pinensis* UQM 2034^T^. Data for strains 2–7 were taken from [11,13,49]. –, Not detected; tr, trace amounts.

Fatty acid	*1*	*2*	*3*	*4*	*5*	*6*	*7*
**Saturated**							
C_14 : 0_	0.8	–	–	1.0	0.5	–	1.0
C_15 : 0_	0.7	–	0.6	–	2.7	–	–
C_16 : 0_	3.5	3.5	0.9	1.9	1.7	2.6	1.9
**Branched**							
iso-C_13 : 0_	0.1	–	4.2	–	0.5	–	1.1
iso-C_13 : 0_ 3-OH	–	–	–	–	–	–	–
iso-C_14 : 0_	–	–	–	–	1.1	–	tr
iso-C_14 : 0_ 3-OH	0.1	–	–	–	–	–	–
C_14 : 0_ 2-OH	0.1	–	–	–	–	–	–
iso-C_15 : 0_	56.7	33.7	34.8	29.7	28.4	26.8	29.0
iso-C_15 : 0_ 3-OH	2.0	2.9	4.0	4.4	2.2	1.3	3.2
anteiso-C_15 : 0_	0.4	1.6	1.7	3.7	0.6	4.9	0.5
C_15 : 0_ 2-OH	0.3	–	0.4	–	0.8	–	–
iso-C_16 : 0_	0.1	–	0.7	–	1.4	1	0.8
iso-C_16 : 0_ 3-OH	0.4	–	1.2	–	0.7	–	–
C_16 : 0_ 2-OH	1.4	–	–	2.6	–	–	–
C_16 : 0_ 3-OH	1.6	2.4	0.9	2.4	1.3	–	2
iso-C_17 : 0_	0.6	–	–	7.4	–	1.6	tr
anteiso-C_17 : 0_	–	–	–	–	–	–	–
iso-C_17 : 0_ 3-OH	15.6	–	16.9	22.7	–	29.4	11.3
C_17 : 0_ 2-OH	0.1	–	0.5	–	–	3.5	–
C_17 : 0_ 3-OH	–	15.5	–	–	15.3	1.2	–
**Unsaturated**					–	–	
C_15 : 1_ ω6*c*	–	–	–	–	–	–	–
iso-C_15 : 1_G	6.1	22.3	8.2	–	26.2	15.6	15.6
anteiso-C_15 : 1_A	–	–	–	–	–	–	tr
C_16 : 1_ ω5*c*	–	–	–	1.7	–	–	6.0
C_17 : 1_ ω6*c*	0.1	–	–	–	–	–	–
iso-C_17 : 1_ ω9*c*	–	–	5.9	–	–	–	–
iso-C_17 : 1_ ω10*c*	–	–	–	–	–	–	–
Summed features*							
1		–			–	–	–
2	0.1	–	–	–	–	–	–
3	7.3	10.6	4.7	13.1	11.2	4.3	23
4	–	–	–	9.4	–	–	–
**Unknown**							
11.543	0.1	–	0.5	–	–	–	–
13.565	1.0	–	8.4	–	1.3	–	–
16.582	1.1	1.2	1.7	–	1.3	1.4	–

*Summed feature 1 is C_13 : 0_ 3-OH and/or iso-C_15 : 1_ h. Summed feature 2 is C_12 : 0_ aldehyde, C_14 : 0_ 3-OH and/or iso-C_16 : 1_I. Summed feature 3 is C_16 : 1_* ω*7*c* and/or iso-C_15 : 0_ 2-OH. Summed feature 4 is iso-C_17 : 1_I and/or anteiso-C_17 : 1_B.

The major polar lipids of strain C216^T^ were phosphatidylethanolamine, three unidentified phospholipids, three unidentified aminophospholipids, one unidentified aminolipid and one unidentified glycolipid (Fig. S3).

Strains C216^T^ and M2295 were positive for utilization of aesculin and *β*-galactosidase and negative for protease activity. Both strains were positive for assimilation of d-glucose, l-arabinose, d-mannose, *N*-acetyl-glucosamine, and maltose. Both strains were negative for indole production. Assimilation of *d*-mannitol, potassium gluconate, capric acid, adipic acid, malic acid, trisodium citrate, and phenylacetic acid was not observed. Neither strain had the ability to reduce nitrates to nitrites or nitrogen. Fermentation of glucose, urease and arginine dehydrogenase activity was not observed under anaerobic conditions.

In conclusion, from the data and observations described, strains C216^T^ and M2295 represent members of a novel genus within the phylum *Bacteroidota*, for which the name *Mycovorax* gen. nov. is proposed, and a novel species within this genus which we propose as *Mycovorax composti* sp. nov.

## Description of *Mycovorax* gen. nov.

*Mycovorax* (My.co.vo’rax. Gr. masc. n. *mykes*, mushroom; L. masc. adj. *vorax*, voracious; N.L. masc. n. *Mycovorax*, devourer of fungi)

Named for the strain’s interaction with *Mycothermus thermophilus*, an ascomycete fungus, where the bacterium degraded the fungal mycelium of *M. thermophilus.* Gram-stain-negative, aerobic, and non-motile. The major fatty acids are iso-C_15 : 0_, iso-C_17 : 0_ 3-OH, C_16 : 1_* ω*7*c*/iso-C_15 : 0_ 2-OH and iso-C_15 : 1_G. The only respiratory quinone is MK-7. Positioned phylogenetically within the phylum *Bacteroidota*. The type species is *Mycovorax composti*.

## Description of *Mycovorax composti* sp. nov.

*Mycovorax composti* (com.pos’ti. N.L. gen. n *composti*, of compost).

Isolated from button mushroom compost. Colonies are orange, 2 mm in size, circular, opaque, and convex with entire margins when grown on 350 Emerson YPSs agar at 45 °C for 2 days. Positive for flexirubin-type pigments. Cells are strictly aerobic, Gram-stain-negative, non-motile and non-spore forming short rods that are 0.8–1.2 µm long and 0.4–0.5 µm wide. Grows at 37–55 °C (optimum, 45–50 °C), at pH 6.5–8.0 (optimum, pH 7.0) and in the presence of 0–2 % (w/v) NaCl (optimum, 0–1 % NaCl). Grows on 350 Emerson YpSs, R2A, TSA and LB. Does not grow on MacConkey agar. Weakly positive for catalase activity and positive for oxidase activity. Indole and H_2_S are not produced. Negative for urease, glucose fermentation, arginine dihydrolase, and protease. Negative for reduction of nitrates to nitrites and nitrates to nitrogen. Positive for chitinase, xylanase, *α-N*-acetylgalactosaminidase, *β*-glucosidase, *β*-galactosidase, and amylase. Hydrolyses gelatin after 48 h. Positive for the assimilation of d-glucose, l-arabinose, d-mannose, maltose, and *N*-acetyl-glucosamine. The only isoprenoid quinone is MK-7. The major fatty acids are iso-C_15 : 0_ (56.7%), iso-C_17 : 0_ 3-OH (15.6%), C_16 : 1_* ω*7*c*/iso-C_15 : 0_ 2-OH (7.3%) and iso-C_15 : 1_G (6.1%). When grown in co-culture with the thermophilic ascomycete *M. thermophilus*, *Mycovorax composti* strain C216^T^ degrades the fungal mycelium and exhibits anti-fungal activity by inhibiting mycelial growth.

Two strains of *Mycovorax composti*, C216^T^ (=DSM 114558^T^=LMG 32998^T^) and M2295 (=DSM 114559=LMG 32997), were isolated from phase II mushroom compost in Australia. The type strain is C216^T^, which was isolated from end-phase II mushroom compost from Victoria, Australia. The DNA G+C content of the type strain is 40.5 mol%. The GenBank accession numbers for the 16S gene sequence and the genomic assembly of strain C216^T^ are OP379917 and CP144143, respectively. Strain M2295 was isolated from end-phase II compost from South Australia, Australia. The GenBank accession number of the 16S gene sequence of strain M2295 is OP379911.

## supplementary material

10.1099/ijsem.0.006496 Uncited Supplementary Material 1.

10.1099/ijsem.0.006496 Uncited Table S1.

## Abbreviations

AAI: average amino acid identity
ANI: average nucleotide identity
CAZy: carbohydrate-active enzymes
CBM: carbohydrate binding module
CE: carbohydrate esterase
COG: clusters of orthologous groups
CTAB: hexadecyltrimethylammonium bromide
dDDH: digital DNA–DNA hybridization
DSMZ: Deutsche Sammlung von Mikroorganismen und Zellkulturen
GH: glycoside hydrolase
GT: glucosyl transferase
GTDB: genome taxonomy database
PL: polysaccharide lyase
RAST: rapid annotations using subsystems technology

## References

[1] Noble R, Gaze RH (1996). Preparation of mushroom (*Agaricus bisporus*) composts in controlled environments: factors influencing compost bulk density and productivity. Int Biodeterior Biodegradation.

[2] Weil JD, Cutter CN, Beelman RB, LaBorde LF (2013). Inactivation of human pathogens during phase II composting of manure-based mushroom growth substrate. J Food Prot.

[3] Straatsma G, Gerrits JPG, Thissen JTNM, Amsing JGM, Loeffen H (2000). Adjustment of the composting process for mushroom cultivation based on initial substrate composition. Bioresour Technol.

[4] Vieira FR, Pecchia JA (2018). An exploration into the bacterial community under different pasteurization conditions during substrate preparation (composting-Phase II) for *Agaricus bisporus* cultivation. Microb Ecol.

[5] Thai M, Safianowicz K, Bell TL, Kertesz MA (2022). Dynamics of microbial community and enzyme activities during preparation of Agaricus bisporus compost substrate. *ISME Commun*.

[6] Jurak E, Gruppen H, Kabel MA, Eggink G, Meyer AS (2015). How mushrooms feed on compost: Conversion of carbohydrates and lignin in industrial wheat straw based compost enabling the growth of *Agaricus bisporus*. PhD Thesis.

[7] Vos AM, Heijboer A, Boschker HTS, Bonnet B, Lugones LG (2017). Microbial biomass in compost during colonization of *Agaricus bisporus*. AMB Express.

[8] Siyoum NA, Surridge K, van der Linde EJ, Korsten L (2016). Microbial succession in white button mushroom production systems from compost and casing to a marketable packed product. Ann Microbiol.

[9] Eichorst SA, Varanasi P, Stavila V, Zemla M, Auer M (2013). Community dynamics of cellulose-adapted thermophilic bacterial consortia. Environ Microbiol.

[10] de Gannes V, Eudoxie G, Hickey WJ (2013). Prokaryotic successions and diversity in composts as revealed by 454-pyrosequencing. Bioresour Technol.

[11] Kämpfer P, Lodders N, Falsen E (2011). *Hydrotalea flava* gen. nov., sp. nov., a new member of the phylum bacteroidetes and allocation of the genera *chitinophaga*, *sediminibacterium*, *lacibacter*, *flavihumibacter*, *flavisolibacter*, *niabella*, *niastella*, *segetibacter*, *parasegetibacter*, *terrimonas*, *ferruginibacter*, *filimonas* and hydrotalea to the family *chitinophagaceae* fam. nov. Int J Syst Evol Microbiol.

[12] Sangkhobol V, Skerman VBD (1981). *Chitinophaga*, a new genus of chitinolytic myxobacteria. Int J Syst Bacteriol.

[13] Siddiqi MZ, Muhammad Shafi S, Choi KD, Im W-T (2016). *Compostibacter hankyongensis* gen. nov., sp. nov., isolated from compost. Int J Syst Evol Microbiol.

[14] Shiratori H, Tagami Y, Morishita T, Kamihara Y, Beppu T (2009). *Filimonas lacunae* gen. nov., sp. nov., a member of the phylum Bacteroidetes isolated from fresh water. Int J Syst Evol Microbiol.

[15] Maeng S, Park Y, Han JH, Lee SE, Kim MK (2021). Pseudocnuella soli gen. nov., sp. nov., a bacterium from soil belonging to the family Chitinophagaceae. Antonie van Leeuwenhoek.

[16] Cooney DG, Emerson R (1964). Thermophilic Fungi: An Account of Their Biology, Activities and Classification.

[17] Rocha Vieira F, Andrew Pecchia J (2021). Fungal community assembly during a high-temperature composting under different pasteurization regimes used to elaborate the *Agaricus bisporus* substrate. Fungal Biol.

[18] Wiegant WM, Wery J, Buitenhuis ET, de Bont JA (1992). Growth-promoting effect of thermophilic fungi on the mycelium of the edible mushroom *Agaricus bisporus*. Appl Environ Microbiol.

[19] Wilson K (2001). Preparation of genomic DNA from bacteria. Curr Protoc Mol Biol.

[20] Sayers EW, Bolton EE, Brister JR, Canese K, Chan J (2022). Database resources of the national center for biotechnology information. Nucleic Acids Res.

[21] Thompson JD, Higgins DG, Gibson TJ (1994). CLUSTAL W: improving the sensitivity of progressive multiple sequence alignment through sequence weighting, position-specific gap penalties and weight matrix choice. Nucleic Acids Res.

[22] Kumar S, Stecher G, Li M, Knyaz C, Tamura K (2018). MEGA X: Molecular Evolutionary Genetics Analysis across computing platforms. Mol Biol Evol.

[23] Afgan E, Baker D, Batut B, van den Beek M, Bouvier D (2018). The Galaxy platform for accessible, reproducible and collaborative biomedical analyses: 2018 update. Nucleic Acids Res.

[24] Wick RR, Judd LM, Gorrie CL, Holt KE (2017). Completing bacterial genome assemblies with multiplex MinION sequencing. Microb Genom.

[25] Filtlong (2017). Github. https://github.com/rrwick/Filtlong.

[26] Lin Y, Yuan J, Kolmogorov M, Shen MW, Chaisson M (2016). Assembly of long error-prone reads using de Bruijn graphs. Proc Natl Acad Sci USA.

[27] Li H, Durbin R (2010). Fast and accurate long-read alignment with Burrows-Wheeler transform. Bioinformatics.

[28] Walker BJ, Abeel T, Shea T, Priest M, Abouelliel A (2014). Pilon: an integrated tool for comprehensive microbial variant detection and genome assembly improvement. PLoS One.

[29] Seemann T (2014). Prokka: rapid prokaryotic genome annotation. Bioinformatics.

[30] (2013). Barrnap 8.0: rapid ribosomal RNA prediction. https://github.com/tseemann/barrnap.

[31] Huerta-Cepas J, Szklarczyk D, Forslund K, Cook H, Heller D (2016). eggNOG 4.5: a hierarchical orthology framework with improved functional annotations for eukaryotic, prokaryotic and viral sequences. Nucleic Acids Res.

[32] Aziz RK, Bartels D, Best AA, DeJongh M, Disz T (2008). The RAST Server: rapid annotations using subsystems technology. BMC Genomics.

[33] Brettin T, Davis JJ, Disz T, Edwards RA, Gerdes S (2015). RASTtk: a modular and extensible implementation of the RAST algorithm for building custom annotation pipelines and annotating batches of genomes. Sci Rep.

[34] Overbeek R, Olson R, Pusch GD, Olsen GJ, Davis JJ (2014). The SEED and the Rapid Annotation of microbial genomes using Subsystems Technology (RAST). Nucleic Acids Res.

[35] Meier-Kolthoff JP, Göker M (2019). TYGS is an automated high-throughput platform for state-of-the-art genome-based taxonomy. Nat Commun.

[36] Dodd D, Cann IKO (2009). Enzymatic deconstruction of xylan for biofuel production. Glob Change Biol Bioenergy.

[37] Walter A, Friz S, Mayer C (2021). Chitin, chitin oligosaccharide, and chitin disaccharide metabolism of *Escherichia coli* revisited: reassignment of the roles of ChiA, ChbR, ChbF, and ChbG. Microb Physiol.

[38] Parks DH, Chuvochina M, Waite DW, Rinke C, Skarshewski A (2018). A standardized bacterial taxonomy based on genome phylogeny substantially revises the tree of life. Nat Biotechnol.

[39] Chaumeil P-A, Mussig AJ, Hugenholtz P, Parks DH (2019). GTDB-Tk: a toolkit to classify genomes with the Genome Taxonomy Database. Bioinformatics.

[40] Price MN, Dehal PS, Arkin AP (2010). FastTree 2–approximately maximum-likelihood trees for large alignments. PLoS One.

[41] Yoon SH, Ha SM, Lim J, Kwon S, Chun J (2017). A large-scale evaluation of algorithms to calculate average nucleotide identity. Antonie van Leeuwenhoek.

[42] Rodriguez-R LM, Konstantinidis KT (2014). Bypassing cultivation to identify bacterial species. Microbe Magazine.

[43] Nicholson AC, Gulvik CA, Whitney AM, Humrighouse BW, Bell ME (2020). Division of the genus *Chryseobacterium:* observation of discontinuities in amino acid identity values, a possible consequence of major extinction events, guides transfer of nine species to the genus *Epilithonimonas*, eleven species to the genus *Kaistella*, and three species to the genus *Halpernia* gen. nov., with description of *Kaistella daneshvariae* sp. nov. and *Epilithonimonas vandammei* sp. nov. derived from clinical specimens. Int J Syst Evol Microbiol.

[44] Richter M, Rosselló-Móra R (2009). Shifting the genomic gold standard for the prokaryotic species definition. Proc Natl Acad Sci USA.

[45] Smibert R (1994). Methods for General and Molecular Bacteriology.

[46] Bernardet J-F, Nakagawa Y, Holmes B (2002). Proposed minimal standards for describing new taxa of the family Flavobacteriaceae and emended description of the family. Int J Syst Evol Microbiol.

[47] Nguyen TM, Kim J (2017). A rapid and simple method for identifying bacterial polar lipid components in wet biomass. J Microbiol.

[48] Jork H, Funk W, Fischer W, Wimmer H (1990). Thin-Layer Chromatography. Reagents and Detection Methods. Physical and Chemical Detection Methods: Fundamentals, Reagents Vol 1a.

[49] Kim B-Y, Weon H-Y, Yoo S-H, Hong S-B, Kwon S-W (2007). *Niabella aurantiaca* gen. nov., sp. nov., isolated from a greenhouse soil in Korea. Int J Syst Evol Microbiol.

[50] Xie C-H, Yokota A (2006). Reclassification of [*Flavobacterium*] *ferrugineum* as *Terrimonas ferruginea* gen. nov., comb. nov., and description of *Terrimonas lutea* sp. nov., isolated from soil. Int J Syst Evol Microbiol.

[51] Weon H-Y, Kim B-Y, Yoo S-H, Lee S-Y, Kwon S-W (2006). *Niastella koreensis* gen. nov., sp. nov. and *Niastella yeongjuensis* sp. nov., novel members of the phylum Bacteroidetes, isolated from soil cultivated with Korean ginseng. Int J Syst Evol Microbiol.

