# Supplementary material for: Mycovorax composti gen. nov., sp. nov., a member of the family Chitinophagaceae isolated from button mushroom compost
Source: Int J Syst Evol Microbiol. 2024 Aug 27;74(8):006496. doi: 10.1099/ijsem.0.006496 (PMC11349053; doi:10.1099/ijsem.0.006496)
Supplement: Uncited Supplementary Material 1. [file ijsem-74-06496-s001.pdf]

Supplementary Materials

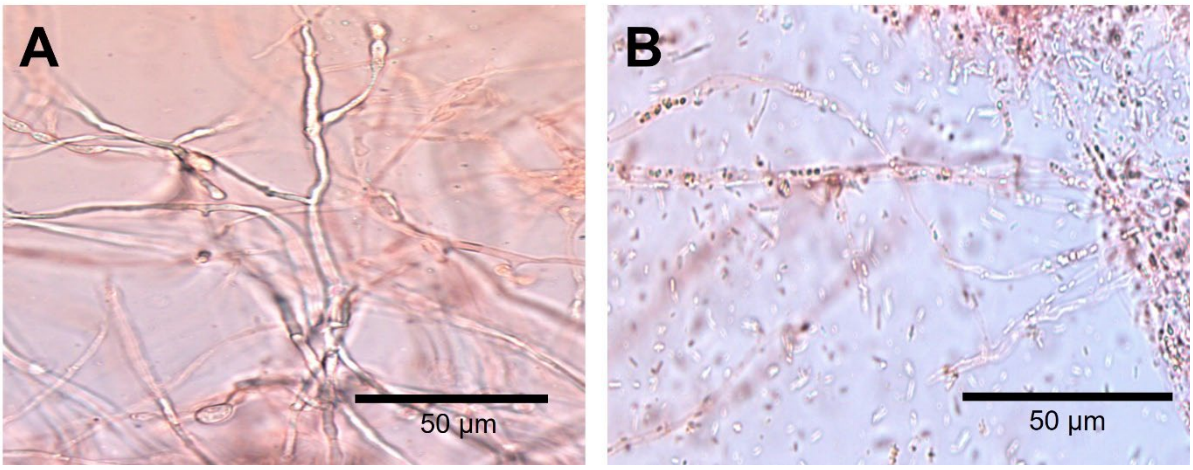

Figure S1. Interaction of strain C216^T with *Mycothermus thermophilus* in YpSs liquid medium. (A) Healthy *M. thermophilus* hyphae in the absence of strain C216^T, (B) *M. thermophilus* hyphae incubated with strain C216^T. Stained with 1% (w/v) Congo Red.

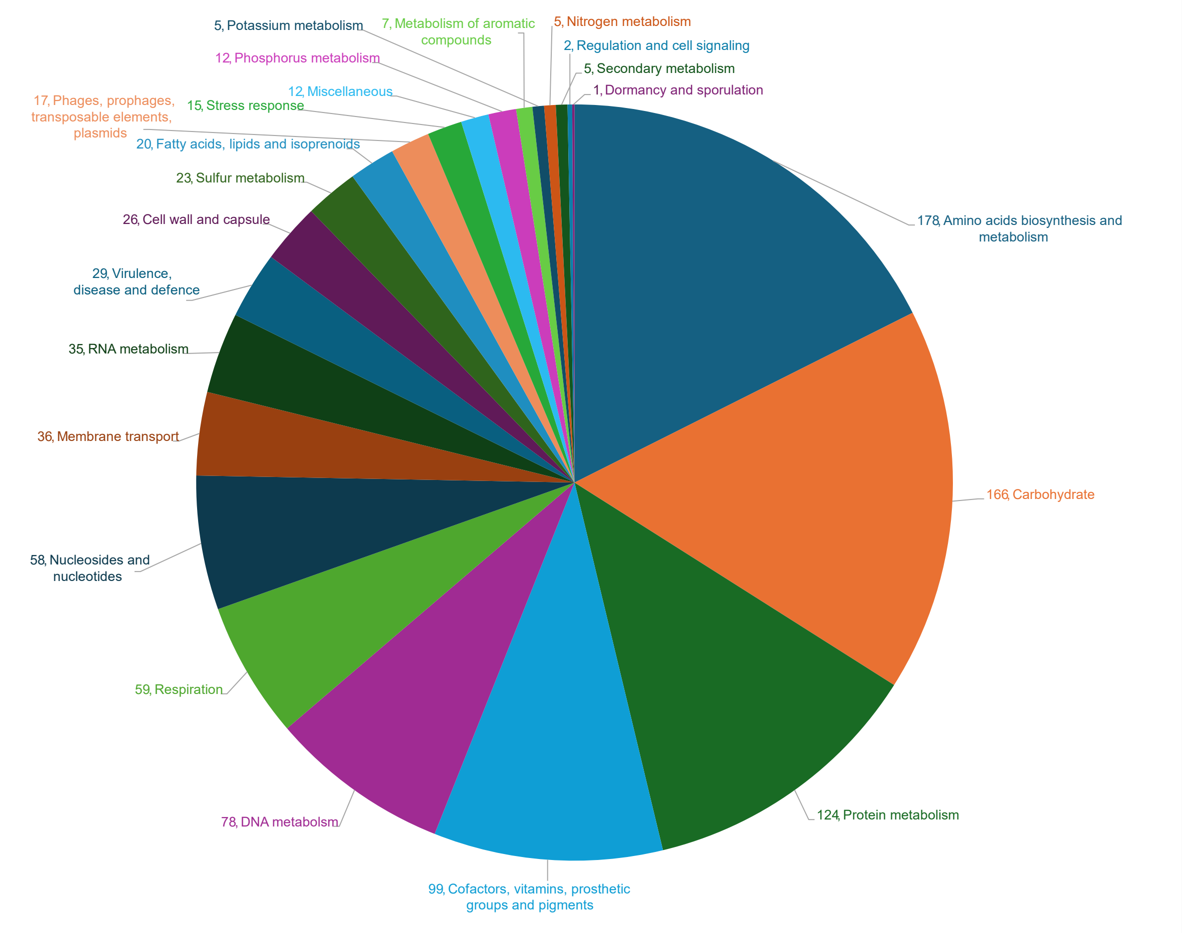

Figure S2. RAST annotation (1, 2) of functional genes (or subsystems) in the genome of strain C216^{T}. Numbers are the feature counts of genes in each subsystem.

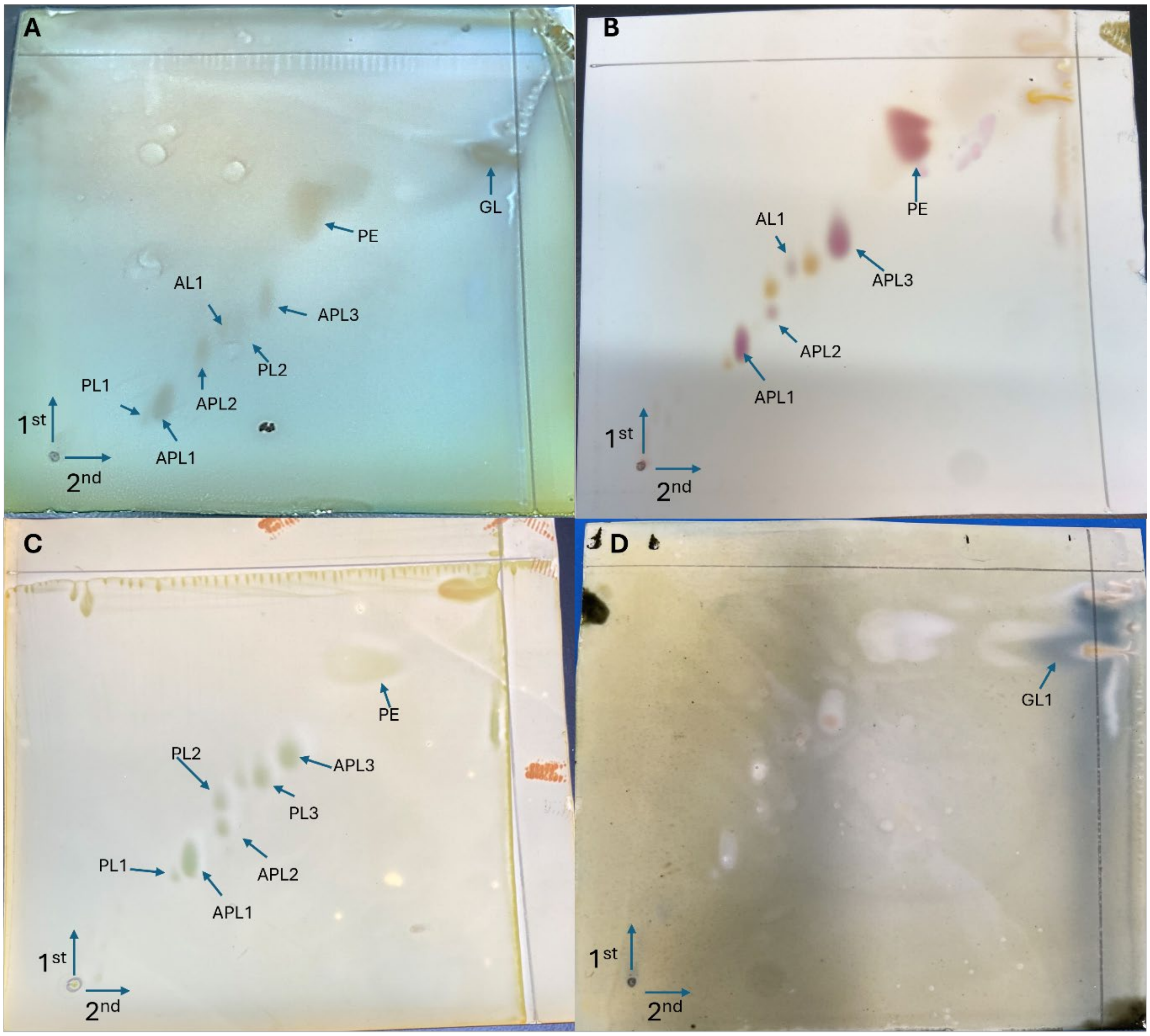

Figure S3. Two-dimensional thin layer chromatography of polar lipids in strain C216^T. A) Total lipids, detected with 5% ethanolic phosphomolybdic acid; B) aminolipids, detected with ninhydrin; C) Phospholipids, detected with Bromthymol blue; D) Glycolipids, detected with diphenylamine. PE: Phosphatidylethanolamine, AL: unidentified aminolipid, APL: unidentified aminophospholipid, PL: unidentified phospholipid and GL: unidentified glycolipid.

Table S1. Genome Features of *Mycovorax composti* C216^{T}**.**

See additional Excel file

Table S2. Percentage of proteins assigned to COG functions and CAZy families of strain C216^T and its six closest phylogenetic neighbours. Data were analysed in Galaxy using the eggNOG package (3, 4).

COG Category	Function	*M. composti* C216^T	*N. aurantiaca* DSM 17617^T	*N. soli* DSM 19437^T	*C. takakiae* RG1-1^T	*T. ferruginea* DSM 30193^T	*P. aquatica* N24^T	*C pinensis* DSM 2588^T
S	Function unknown	22.28%	22.51%	22.58%	23.55%	23.40%	23.76%	21.36%
G	Carbohydrate transport and metabolism	8.93%	10.99%	10.54%	7.95%	6.17%	7.74%	6.85%
M	Cell wall/ membrane/ envelope biogenesis	7.82%	8.90%	7.97%	8.86%	9.32%	8.93%	7.91%
E	Amino acid transport and metabolism	7.30%	6.76%	7.41%	6.70%	6.53%	6.78%	5.69%
P	Inorganic ion transport and metabolism	6.75%	7.37%	6.30%	5.77%	5.18%	5.97%	6.49%
J	Translation, ribosomal structure and biogenesis	6.08%	4.18%	4.80%	4.69%	5.04%	5.20%	3.20%
K	Transcription	5.93%	6.85%	6.39%	5.40%	6.59%	4.59%	10.77%
-	Unassigned	5.93%	8.17%	7.29%	8.46%	8.22%	7.72%	9.41%
C	Energy Production and conversion	5.19%	4.91%	5.48%	5.20%	5.01%	5.86%	5.52%
L	Replication, recombination and repair	4.56%	3.71%	3.59%	5.08%	3.60%	4.78%	3.90%
H	Coenzyme transport and metabolism	4.52%	3.64%	4.18%	3.88%	3.75%	3.68%	3.73%
I	Lipid Transport and metabolism	3.82%	3.29%	3.56%	3.63%	4.31%	3.73%	4.18%

O	Posttranslational modification, protein turnover, chaperones	3.71%	3.33%	3.70%	3.36%	4.08%	4.04%	3.72%
F	Nucleotide transport and metabolism	2.71%	2.02%	2.29%	2.01%	2.06%	2.13%	1.72%
T	Signal transduction mechanisms	2.67%	3.87%	3.67%	6.18%	5.80%	4.92%	6.53%
Q	Secondary metabolites biosynthesis, transport and catabolism	2.26%	2.44%	2.01%	1.77%	2.56%	1.85%	3.10%
U	Intracellular trafficking, secretion and vesicular transport	2.08%	1.64%	1.67%	2.33%	2.00%	2.02%	1.48%
V	Defence mechanisms	1.63%	1.90%	2.29%	1.69%	2.22%	2.05%	2.21%
D	Cell cycle control, cell division, chromosome partitioning	1.07%	0.89%	0.90%	1.18%	1.15%	1.19%	0.71%
N	Cell motility	0.52%	0.66%	0.59%	0.47%	0.93%	0.55%	0.77%
Z	Cytoskeleton	0.19%	0.14%	0.06%	0.05%	0.17%	0.11%	0.08%
X	Mobilome: prophages and transposons	0.00%	0.00%	0.00%	0.00%	0.00%	0.00%	0.00%
Y	Nuclear structure	0.00%	0.00%	0.00%	0.00%	0.00%	0.00%	0.00%
A	RNA processing and modification	0.00%	0.00%	0.00%	0.00%	0.00%	0.00%	0.02%
B	Chromatin structure and dynamics	0.00%	0.00%	0.00%	0.05%	0.03%	0.03%	0.05%
W	Extracellular structures	0.00%	0.00%	0.00%	0.22%	0.08%	0.08%	0.02%

**CAZy**								
CBM	Carbohydrate Binding Modules	3.94%	2.49%	2.94%	7.11%	7.95%	3.39%	9.29%
CE	Carbohydrate Esterases	6.30%	1.66%	3.92%	6.60%	7.95%	10.17%	5.00%
GH	Glycoside Hydrolases	57.48%	73.44%	73.04%	51.27%	53.98%	49.15%	56.43%
GT	Glucosyl Transferases	28.35%	19.09%	16.67%	32.99%	28.41%	32.20%	28.21%
PL	Polysaccharide Lyases	3.94%	3.32%	3.43%	2.03%	1.70%	5.08%	1.07%

References

1. Aziz RK, Bartels D, Best AA, DeJongh M, Disz T, Edwards RA, et al. The RAST Server: rapid annotations using subsystems technology. BMC Genomics. 2008;9:Article 75. doi: 10.1186/1471-2164-9-75.
2. Brettin T, Davis JJ, Disz T, Edwards RA, Gerdes S, Olsen GJ, et al. RASTtk: a modular and extensible implementation of the RAST algorithm for building custom annotation pipelines and annotating batches of genomes. Sci Rep. 2015;5:Article 8365. doi: 10.1038/srep08365.
3. Huerta-Cepas J, Szklarczyk D, Forslund K, Cook H, Heller D, Walter MC, et al. eggNOG 4.5: a hierarchical orthology framework with improved functional annotations for eukaryotic, prokaryotic and viral sequences. Nucleic Acids Res. 2015;44:D286-D293. doi: 10.1093/nar/gkv1248.
4. Afgan E, Baker D, Batut B, van den Beek M, Bouvier D, Čech M, et al. The Galaxy platform for accessible, reproducible and collaborative biomedical analyses: 2018 update. Nucleic Acids Res. 2018;46:537-544. doi: https://doi.org/10.1093/nar/gky379.
